# PPAR*γ* in Neuroblastoma

**DOI:** 10.1155/2008/917815

**Published:** 2008-05-28

**Authors:** Alessandro Peri, Ilaria Cellai, Susanna Benvenuti, Paola Luciani, Silvana Baglioni, Mario Serio

**Affiliations:** Endocrine Unit, Department of Clinical Physiopathology, Center for Research, Transfer and High Education on Chronic, Inflammatory, Degenerative and Neoplastic Disorders (DENOThe), University of Florence, 50139 Florence, Italy

## Abstract

Neuroblastoma (NB) is the most common extracranial tumor in children and accounts for around 15% of all paediatric oncology deaths. The treatment of NB includes surgery, chemotherapy, and radiotherapy. Unfortunately, most children with NB present with advanced disease, and more than 60% of patients with high-risk features will have a poor prognosis despite intensive therapy. Agonists of the nuclear receptor peroxisome proliferator-activated receptor *γ* (PPAR*γ*) have been shown to have pleiotropic effects, including antineoplastic effects. The studies that addressed the role and the possible mechanism(s) of action of PPAR*γ* in NB cells are reviewed.

## 1. INTRODUCTION

Neuroblastoma (NB), the most common extracranial solid tumor in children, accounts for more than 7% of malignancies in patients younger than 15 years and around 15% of all paediatric oncology deaths [1]. The disease has a heterogeneous clinical presentation and course [2]. First of all, NB is a disease of the sympaticoadrenal lineage of the neural crest, and therefore tumors can develop anywhere in the sympathetic nervous system. The majority of NB is developed within the abdomen and at least 50% of these tumors arise in the adrenal medulla [2]. Other frequent localizations include the neck, chest, and pelvis [3]. The clinical presentation of the disease may be also highly variable and depends on the site of the primary tumor as well as on the presence or absence of metastatic disease (mostly haematogeneous dissemination to cortical bone, bone marrow, liver, and noncontiguous lymph nodes) or paraneoplastic syndromes. The diagnosis of NB is based on histopathological assessment of tumor tissue or on the detection of cancer cells in a bone marrow aspirate/biopsy, together with the presence of increased levels of urinary catecholamines [2]. Imaging studies for the localization of the disease include computed tomography, magnetic resonance, ^99^mTc-diphosphonate, or metaiodobenzylguanidine (using ^123^I) scintigraphy for the detection of bone metastases.

The treatment of NB includes surgery, chemotherapy (i.e., cisplatin, etoposide, doxorubicin, cyclophosphamide, vincristine) [4], and radiotheraphy. Unfortunately, although substantial improvement in outcome of certain subsets of patients has been observed during the past few decades [2], most children with NB present with advanced disease and more than 60% of patients with high-risk features will have a poor prognosis despite intensive therapy [5, 6]. Thus, research efforts to understand the biological basis of NB and to identify new and more effective therapies are essential to improve the outcome for these children. In the last years an expanding number of new agents have been developed for use in high-risk patients affected by recurrent disease. Cytotoxic agents, such as the topoisomerase 1 inhibitors topotecan and irinotecan, have an acceptable toxicity profile and are effectively used in early relapsing NB [7–10]. The delivery of radioactive molecules that are selectively concentrated in NB cells, such as metaiodobenzylguanidine, somatostatin analogues, anti-G_D2_ (a disialoganglioside) antibodies, has been used in clinical trials [11–22]. G_D2_-targeted therapies using monoclonal antibodies are under investigation in phase III trials [19, 23, 24], and other immunotherapeutic strategies (i.e., vaccination or cellular immunotherapy using engineered cytolityc T lymphocytes) are currently investigated [25, 26]. Similarly, angiogenesis [27–33] and tyrosine kinase [34–38] inhibitors appear as an attractive therapeutic option and clinical trials are ongoing. Retinoids have been shown to interfere with cell growth and to induce apoptosis in NB cells [39, 40] and preliminary clinical trials with retinoids in NB resulted in improved event-free survival in high-risk patients, with limited toxic effects [41, 42]. Thiazolidinediones (TZDs) are a class of molecules that activate the nuclear receptor peroxisome proliferator-activated receptor *γ* (PPAR*γ*) [43] and promote association with the 9-*cis* retinoic X receptor (RXR) to form functional heterodimers that recognize its cognate DNA response element within target genes [44, 45]. TZDs have been shown to have antineoplastic effects, as extensively discussed in this issue of the journal, in agreement with the demonstration that PPAR*γ*/RXR signalling exerts an important role in inhibiting cell proliferation and/or in inducing apoptosis [46]. It has been also shown that PPAR*γ* and RXR ligands may have a synergistic effect in inducing cell differentiation [47, 48] and in inhibiting cell growth in different tumors, such as colon, lung, and breast cancer [49–51]. There is evidence that also PPAR*α* and PPAR*β* ligands may play a role in counteracting tumoral cell growth and in promoting cell differentiation, including neuroblastoma cells [52, 53]. However, most of the reports covering this issue, that have been published in the literature so far, deal with PPAR*γ* agonists. Therefore, the role of PPAR*γ* ligands as a possible therapeutic option in NB is reviewed and discussed here.

## 2. PPAR*γ* AND PPAR*γ* AGONISTS IN NEUROBLASTOMA

The first demonstration that PPAR*γ* is expressed in NB cells was provided by Han et al. in 2001 [54]. Using RT-PCR the authors showed that LA-N-5 NB cells express also PPAR*β*, but not PPAR*α*. Similarly, in sections from human primary NB immunostaining for PPAR*γ* was detected in the nucleus and occasionally in the cytoplasm of cells, particularly in those showing ganglionic differentiation. Sato et al. [55] addressed the possibility that the amount of expression of PPAR*γ* in NB might be correlated to patients' outcome. To this purpose, the level of mRNA was measured by semiquantitative RT-PCR in NB samples from 17 patients under the age of one year. In this subset of patients, spontaneous differentiation and regression are often observed [56], and some investigators suggested to observe these patients without surgery until there is an increase of vanilmandelic acid (VMA) or tumoral growth occurs [57, 58]. PPAR*γ* mRNA was present in 12 samples. No difference between the expression of PPAR*γ* and histology, age, staging, DNA ploidy was observed, yet a correlation with the change in urinary VMA was found. In fact, in samples resected from patients, who showed a reduction of VMA in the period of time preceding surgery (2–7 months), higher PPAR*γ* expression was detected compared to those patients in which VMA increased. The authors hypothesized that PPAR*γ* might play a role in the decrease of VMA and hence in the regression of early-onset NB. Thereafter, several studies addressed the potential role of endogenous or synthetic PPAR*γ* ligands in counteracting NB cell growth.

5-*Deoxy*-Δ^12,14^-*prostaglandin J_2_* (15-deoxy-PGJ_2_) is a naturally occurring downstream metabolite of PGD_2_, that is produced by degradationof PGD_2_ [59]. In contrast to classic prostaglandins, which act after binding to cell surface G-protein coupled receptors(GPCRs), 15-deoxy-PGJ_2_ is a high-affinity endogenous ligand of PPAR*γ*. A pro-apoptotic effect of 15-deoxy-PGJ_2_ in SH-SY5Y NB cells, that was reverted by the caspase inhibitor Z-VAD, was reported by Rohn et al. [60]. A subsequent study confirmed that 15-deoxy-PGJ_2_ was able to inhibit cell growth and to induce apoptosis via the activation of ERK2 in two additional NB cell lines (i.e., SK-N-SH and SK-N-MC). An increase of the expression of the pro-apoptotic proteins caspase-3, caspase-9, and Bax, together with the decrease of the anti-apoptotic protein Bcl-2, was also observed [61]. The PPAR*γ* antagonist GW9662 reverted the effects of 15-deoxy-PGJ_2_, including the activation of ERK2. The authors concluded that 15-deoxy-PGJ_2_ induced apoptosis in a PPAR*γ*-dependent manner through the activation of ERK pathway. Another study showed that the mechanism by which 15-deoxy-PGJ_2_ arrests cell growth may vary depending on the content of lipids in the culture medium [62]. In particular, the delipidation of fetal calf serum, which removes known serum lipid mitogens including lysophosphatidic acid [63] and sphingosine 1-phosphate [64], potentiated the degree of 15-deoxy-PGJ_2_-induced growth inhibition via PPAR*γ*-dependent apoptosis in the NB cell line IMR-32. Conversely, growth inhibition in the presence of complete medium occurred through programmed cell death typeII (autophagy).

PPAR*γ*-independent effects of 15-deoxy-PGJ_2_ have been also described. Jung et al. reported that this PPAR*γ* ligand was able to increase NGF-induced differentiation of PC-12 NB cells, as assessed by neurite extension and expression of neurofilament [65]. Pretreatment with the PPAR*γ* antagonist bisphenol A diglycidyl either did not alter the differentiating activity of 15-deoxy-PGJ_2_. The fact that PC-12 cells do not express PPAR*γ* further supported the hypothesis that the biological effects elicited by 15-deoxy-PGJ_2_ were not mediated by this receptor. Conversely, 15-deoxy-PGJ_2_ enhanced NGF-induced p38 MAP kinase expression and phosphorylation as well as the activation of transcription factor AP-1, that on turn were counteracted by a specific inhibitor of p38 MAP kinase (SB203580). Altogether, these data suggested that the promoting effect of 15-deoxy-PGJ_2_ on cell differentiation may be mediated by the activation of p38 MAP kinase in conjunction with the AP-1 signalling pathway.

Other studies addressed the role of *synthetic PPARγ ligands* in counteracting cell growth in NB. In the already mentioned work by Han et al., in which the presence of PPAR*γ* in NB cells was described for the first time, the authors also demonstrated that the synthetic PPAR*γ* agonist GW1929 induced the differentiation of LA-N-5 cells and inhibited cell proliferation [54]. A subsequent study of the same group showed that the prodifferentiating effect of GW1929 is mediated by PPAR*γ*, because it was inhibited by the cotreatment with specific antagonists [66]. The antiproliferative effects of the TZDs ciglitazone, pioglitazone, troglitazone, and rosiglitazone in different NB cell lines (i.e., LAN-1, LAN-5, LS, IMR-32, SK-N-SH, SH-SY5Y) were determined by Valentiner et al. [67]. In these cell lines, which express PPAR*γ*, the four ligands were able to markedly inhibit cell growth at the highest doses that were used (10 and 100 *μ*M). Ciglitazone determined the strongest inhibitory effect (more than 90% inhibition). The potency of the different PPAR*γ* ligands was not related to the amount of expression of PPAR*γ* in NB cell lines. Thus, the authors hypothesized that the effects of the molecules that were used seem to be independent of the amount of PPAR*γ* protein in one particular cell line. Conversely, they concluded that the response to PPAR*γ* ligands may rather depend on various cellular conditions, which are associated with the function of the receptor, such as its activation, translocation to the nucleus and binding to PPAR response elements (PPRE). The role played by PPAR*γ* transactivation was confirmed by the finding that growth inhibition determined by 15-deoxy-PGJ_2_ and ciglitazone in NB cells was counteracted by the repression of PPAR*γ* transactivation via retinoblastoma protein overexpression [68]. Further studies investigated whether the inhibitory effect of TZDs on cell growth was mediated, at least partially, by a stimulatory effect on apoptosis. Kato et al. found that in NB-1 cells troglitazone induced PPAR*γ*-dependent apoptosis [69]. Similar data were reported later on by Schultze et al. [70], who showed that in SHEP NB cells the pro-apoptotic effect of the death ligand TRAIL is reinforced by troglitazone. However, troglitazone-induced sensitization to TRAIL appeared to be PPAR*γ*-independent, because it was achieved at concentrations that failed to activate PPAR*γ*. Conversely, the authors highlighted the fact that troglitazone may induce apoptotic death by various PPAR*γ*-independent mechanisms. In particular, troglitazone led to a marked downregulation of the antiapoptotic protein Survivin, as well as to an upregulation of the agonistic TRAIL receptor TRAIL-R2.

Overall, these data strongly indicate that PPAR*γ* ligands are able to effectively counteract cell growth and to induce apoptosis in NB cells. Undoubtedly, the role of PPAR*γ* in eliciting these responses would be further clarified by studies designed for instance to manipulate gene expression (i.e., by small interfering RNA or dominant negative strategies). To our knowledge, there are only two reports from one Korean group showing, in contrast to the current opinion, that a PPAR*γ* agonist (i.e., rosiglitazone) protects NB (SH-SY5Y) cells against the neurotoxins acetaldehyde and 1-methyl-4-phenylpyridinium ion, through inhibition of apoptosis [71, 72].

## 3. DIFFERENTIAL PPAR*γ* TRANSACTIVATION IN NEUROBLASTOMA CELL LINES WITH A DIFFERENT PHENOTYPE: RELATIONSHIP WITH THE RESPONSE TO ROSIGLITAZONE

NB is a phenotypically heterogeneous tumor, displaying cells of neuronal, melanocytic, or glial/schwannian lineage. This cellular heterogeneity is also present in vitro, where cells of neuroblastic (N) or stromal (S) type may be identified. It has been hypothesized that the sensitivity to PPAR*γ* ligands may be, at least partially, dependent on the different cell phenotype. To this purpose, Servidei et al. examined the response of 8 different NB cell lines with N (SH-SY5Y, LA-N-5, SMS-KCNR, SK-N-DZ), mixed (SK-N-FI, LA-N-1), or S (SH-EP1, SK-N-AS) phenotype to PPAR*γ* agonists [73]. All the cell lines investigated expressed a functionally active PPAR*γ*. 15-deoxy-PGJ_2_ and rosiglitazone inhibited cell growth in all cell lines, and the sensitivity appeared to be more related to the cell phenotype than to PPAR*γ* expression. In particular, the N type cells appeared the most sensitive to treatment. In this experimental setting, the cotreatment with PPAR*γ* ligands and the RXR ligand 9-*cis* retinoic acid did not determine any synergistic effect on growth inhibition. The more evident response of N type cells to PPAR*γ* ligands was in part related to their higher capability to undergo apoptosis, although only 15-deoxy-PGJ_2_ appeared to effectively induce the apoptotic cascade in these cells. It has to be said that in this study some experimental observations (i.e., apoptosis and cell viability) were not performed in all the investigated NB cell lines.

In order to further clarify the mechanisms underlying the response of NB cells to PPAR*γ* agonists, we compared the response of two cell lines (SH-SY5Y, N type, and SK-N-AS, S type) to rosiglitazone. In contrast to the above-mentioned findings, we observed that micromolar concentrations of rosiglitazone inhibited cell proliferation and reduced cell viability more effectively in SK-N-AS than in SH-SY5Y [74]. The PPAR*γ* antagonist BADGE reverted the effect of rosiglitazone, thus suggesting a direct role of PPAR*γ* in mediating the effects of this agonist on cell proliferation and viability. In addition, we found that SK-N-AS cells were more sensitive to rosiglitazone in terms of reduction of cell adhesion and invasiveness. The latter effect was in agreement with rosiglitazone-dependent reduced expression of matrix metalloproteinase-9 (MMP-9). In addition, rosiglitazone determined a trend toward increased expression levels of tissue inhibitor of matrix metalloproteinase-1 (TIMP-1). MMPs, which promote the invasion of extracellular matrix by tumoral cells, have been related to the progression of different tumors, including NB [75, 76]. In our study, we also addressed the possible role of rosiglitazone in inducing apoptosis. We demonstrated that micromolar concentrations of this molecule were able to induce caspase-3 activation in SK-N-AS, but not in SH-SY5Y (up to 50 *μ*M). Therefore, all our data indicated that rosiglitazone played an effective antitumoral role in the S type SK-N-AS, yet not in the N type SH-SY5Y NB cells. Although our study was limited to two cell lines, this apparent prevalent effect on a particular cell phenotype may have clinical resonance. In fact, it is known that in NB, following cytotoxic therapy, the residual tumor often shows a reduction of the neuroblastic elements and the persistence of stromal components [77]. Hence, a molecule that appears to have S type NB cells as a preferential target might be of interest in the setting of residual disease.

**Figure 1:**
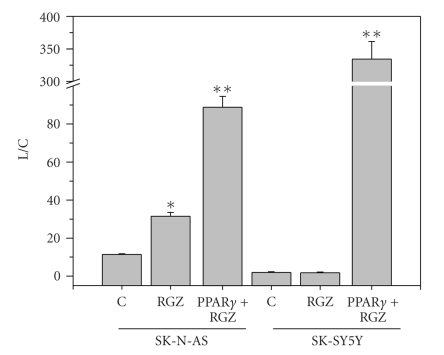
PPAR*γ* transcriptional activity in control untreated NB cells (C), in cells treated with rosiglitazone (RGZ) (20 *μ*M), and in cells transfected with PPAR*γ* and treated with RGZ. L/C: peroxisome proliferator response element-n7_3_-tk-luciferase reporter activity, normalized for CAT activity. * = *P* < 0.05 *versus* C. ** = *P* < 0.05 *versus* C, and *versus* RGZ-treated cells, in the absence of PPAR*γ* transfection (from [74], modified).

**Figure 2:**
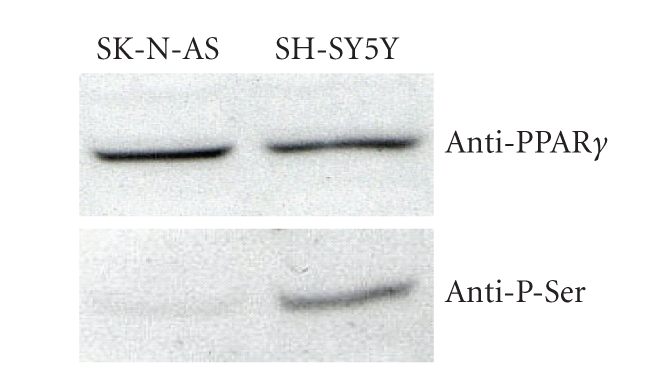
Detection of total (anti-PPAR*γ* antibody) and phosphorylated (anti-P-Ser antibody) PPAR*γ*, by Western blot analysis after PPAR*γ* immunoprecipitation (from [74], modified).

A further aim of our study was to determine the reason underlying the peculiar sensitivity to rosiglitazone displayed by SK-N-AS cells. Both SK-N-AS and SH-SY5Y expressed a similar amount of PPAR*γ*. However, in transient transfection experiments, in which a PPRE-thimidine kinase luciferase reporter plasmid was inserted, we observed that in SK-N-AS 20 *μ*M rosiglitazone induced a near three-fold increase of the reporter activity compared to untreated cells. Conversely, no effect was elicited in SH-SY5Y. Only when these cells were co-transfected with a human PPAR*γ* expression plasmid, the response to rosiglitazone was present. These data indicated that the original lack of response showed by SH-SY5Y was due to a very low or absent transactivation potential of the endogenous PPAR*γ* (Figure 1). The different efficacy of PPAR*γ* as a transcriptional activator in the two cell lines might be hypothetically due to the presence of a PPAR*γ* gene mutation. However, no mutation was found in the entire coding region of the gene. Conversely, we found that the amount of phosphorylated PPAR*γ* was markedly lower in SK-N-AS than in SH-SY5Y cells (Figure 2). There is evidence that phosphorylation reduces the activity of the receptor [78]. Therefore, our conclusion was that the higher efficacy of rosiglitazone in SK-N-AS cells was due to a reduced phosphorylation status, hence to increased activity, of PPAR*γ*. To our knowledge, this was the first demonstration that the response of NB cells to TZDs may be dependent on PPAR*γ* transactivation.

## 4. PPAR*γ* AGONISTS IN NEUROBLASTOMA XENOGRAFT MODELS

To our knowledge, no study on the in vivo effect of TZDs in neuroblastoma has been published so far. However, our very recent preliminary in vivo observations on CD-1 athymic nude mice, in which SK-N-AS cells were subcutaneously inoculated, appear to confirm our previous in vitro observations [74]. Rosiglitazone (150 mg/kg/day, in agreement with the average dose used in other in vivo studies addressing different tumors) was administered by gavage for 4 weeks. Tumoral growth was markedly reduced compared to control mice, treated with the vehicle alone. At the end of treatment, the weight of the tumor in rosiglitazone-treated animals was about 60% less than in control animals [Cellai et al; unpublished data]. An extensive molecular characterization of tumor specimens is currently ongoing, in order to elucidate the mechanisms underlying the growth inhibitory effect of rosiglitazone observed in vivo in our xenograft model.

## 5. CONCLUSIONS

In the last few years in vitro studies have shown that PPAR*γ* agonists may inhibit NB cell growth by stimulating cell differentiation and/or by inducing apoptosis. The different molecules that have been tested have generally produced similar results. However, the mode of action may change depending on the agonist and/or on the different cell line used. In addition, both PPAR*γ*-dependent as well as PPAR*γ*-independent effects have been described. Our recent data suggest that PPAR*γ* transactivation, determined at least in part by the phosphorylation status of the receptor, may play an important role in determining the response of NB cells to PPAR*γ* agonists. However, the exact mechanisms of action and the possibility to predict the success or failure of the treatment of NB with these molecules are, at this time, matter of further in vitro as well as in vivo research.
